# Time to Task Failure and Motor Cortical Activity Depend on the Type of Feedback in Visuomotor Tasks

**DOI:** 10.1371/journal.pone.0032433

**Published:** 2012-03-09

**Authors:** Benedikt Lauber, Christian Leukel, Albert Gollhofer, Wolfgang Taube

**Affiliations:** 1 Department of Sport Science, University of Freiburg, Freiburg, Germany; 2 Department of Medicine, Movement and Sports Science, University of Fribourg, Fribourg, Switzerland; University Medical Center Groningen UMCG, The Netherlands

## Abstract

The present study aimed to elucidate whether the type of feedback influences the performance and the motor cortical activity when executing identical visuomotor tasks. For this purpose, time to task failure was measured during position- and force-controlled muscular contractions. Subjects received either visual feedback about the force produced by pressing a force transducer or about the actual position between thumb and index finger. Participants were instructed to either match the force level of 30% MVC or the finger position corresponding to the thumb and index finger angle at this contraction intensity. Subjects demonstrated a shorter time to task failure when they were provided with feedback about their joint position (11.5±6.2 min) instead of force feedback (19.2±12.8 min; P = 0.01). To test differences in motor cortical activity between position- and force-controlled contractions, subthreshold transcranial magnetic stimulation (subTMS) was applied while executing submaximal (20% MVC) contractions. SubTMS resulted in a suppression of the first dorsal interosseus muscle (FDI) EMG in both tasks. However, the mean suppression for the position-controlled task was significantly greater (18.6±9.4% vs. 13.3±7.5%; P = 0.025) and lasted longer (13.9±7.5 ms vs. 9.3±4.3 ms; P = 0.024) compared to the force-controlled task. The FDI background EMG obtained without stimulation was comparable in all conditions. The present results demonstrate that the presentation of different feedback modalities influences the time to task failure as well as the cortical activity. As only the feedback was altered but not the mechanics of the task, the present results add to the body of evidence that suggests that the central nervous system processes force and position information in different ways.

## Introduction

Sensory feedback is crucial when performing motor tasks. One of the various types of sensory feedback, which can be provided during a motor task, is referred to as augmented external feedback. Augmented external feedback adds additional sensory information from an external source (e.g. visually displayed force levels or information about the joint position) and therefore provides a quantified knowledge of the performance of a motor task [1]. Recent studies have proposed that augmented visual feedback presented during task execution (referred to as online feedback) is crucial for inducing automatic visuomotor adaptations [2], [3]. Furthermore, it has been shown that online augmented feedback was important to accomplish bimanual coordination patterns [4], [5], [6], [7], [8]. On a very functional level, previous motor learning studies showed that visually displayed feedback can facilitate the learning of a golf shot [9] and increase the power output during leg press exercise [10]. Besides the finding that augmented feedback is able to facilitate performance it is unknown whether certain feedback modalities facilitate more than others.

Previously, Milner and Hinder [11] have suggested that position rather than force information is used when adapting to changes in environmental dynamics. In this study, subject learned to move a handle from a start to an end point in a force field where a spring produced a lateral force to the target hand path (position-dependent force field; PF). Occasionally, the position-dependent force field was doubled (PF 2) but in both cases (PF and PF2), subjects were able to reduce their lateral error after only 1 trial. When the strong force field (PF2) returned to its initial magnitude (PF), a strong aftereffect was apparent. However, this aftereffect was abolished if the second PF2 trial was replaced by an oppositely directed velocity-dependent force field (VF). Interestingly, in the following VF trials, subjects did not rapidly adapt to this force-field condition like in the PF and PF2 conditions but continued to produce a force, which assisted the new direction of the force field for approximately 15 trials. Therefore, the authors concluded that the CNS uses position information rather than force information to adapt to changes in environmental dynamics. On a behavioral level, the results by Milner and Hinder [11] indicate that there exist differences for the integration of force and position information. However, what remains unanswered is whether there also exist differences in the integration of position and/or force information when the environmental dynamics of the tasks are identical. Accordingly, the idea of the present study was to ask subjects to perform identical tasks but provide them with different feedback: a) visually displayed feedback about the force they produced or b) visually displayed feedback about the position of their fingers. The task consisted of pressing a hand gripper with a spring like behavior so that changes in the position of the hand gripper where proportional to changes of the force level. Thus, subjects received either feedback about their joint position or their exerted force while they had to maintain a certain predefined level by pressing a hand gripper. Two experimental protocols were conducted: In protocol 1, subjects were tested in a fatiguing task where time to task failure was determined during position and force-controlled contractions. The results showed that the time to task failure was significantly reduced when subjects received feedback on joint position compared to the condition when feedback on the exerted force was given (for details see result section). As the mechanics of the two tasks were identical, it is reasonable to assume that the differences in time to task failure can be attributed to different motor control strategies. Therefore, the second part of our study (protocol 2) tested the hypothesis that the motor cortex (M1) provides a neural source, which differentially integrates the position and force feedback in order to generate accurate movement corrections. M1 is a prime candidate because (1) it integrates and processes afferent information as being part of the transcortical (reflex) loop [12], [13]; and (2) represents a key junction for voluntary control which incorporates information about of the limbs when generating goal directed voluntary actions [14], [15].

To test the assumption that the motor cortex is differently activated with respect to changes in the feedback modality, subthreshold TMS (subTMS) was applied to the motor cortex during the execution of the position and force-controlled contraction (Protocol 2). Davey et al. [16] were the first to demonstrate that a single transcranial magnetic stimulus below the threshold to elicit an MEP can evoke a suppression of the EMG of a voluntarily contracted muscle without prior facilitation. A number of control experiments suggested that this TMS-evoked EMG suppression derives from the activation of intracortical inhibitory interneurons, which suppress and thereby reduce the output from the motor cortex [16]–[18]. Furthermore, it was shown that this EMG suppression is of cortical origin as transcranial electric stimulation (TES), which is thought to stimulate the corticospinal axons directly, failed to suppress the EMG [17]. Thus, decreases in the muscular activity after subTMS were argued to indicate direct cortical contributions to the task. This was supported by a study of Di Lazzaro et al. [19] showing that such low stimulation intensities which suppress EMG activity are below intensities to evoke recognizable spinal cord volleys measured with epidural electrodes in conscious subjects. Furthermore, a recent study of Petersen et al. [20] showed that voluntary drive seems to be a prerequisite to cause an EMG suppression. They showed that subTMS caused a suppression of the ongoing EMG only during voluntary breathing whereas the EMG suppression was only marginal during involuntary breathing. In the current study, subTMS was used to identify differences in motor cortical processing with respect to the type of feedback.

## Materials and Methods

### Subjects

In protocol 1, ten healthy subjects (5 woman and 5 men, 27±2.7 years) participated. For protocol 2, additional ten healthy subjects (2 women and 8 men, 26±1.9 years) volunteered to participate. According to the Oldfield handedness inventory [21], all subjects were right handed and gave their written informed consent to the experiment which was approved by the ethics committee of the University of Freiburg and in accordance with the Declaration of Helsinki.

### Mechanical recording

**Figure 1:**
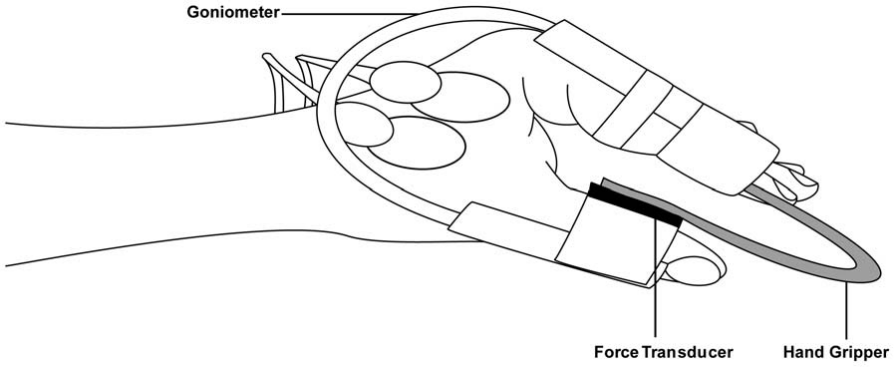
Schematic drawing of the experimental setup. The subjects held the hand gripper between thumb and index finger. The force transducer was taped on the inside of the thumb to register the exerted force and the goniometer was taped on thumb and index finger to register movements of the two fingers. The hand gripper had spring like properties, i.e. the more the force increased the more the two handles were approached to each other.

Subjects were seated in an upright position in an adjustable chair facing a 21-inch monitor placed 1 m in front of them. The non-dominant left hand was used in all subjects while the subject's shoulder was abducted so that the forearm could rest on the table placed to their side. A custom-built “hand gripper” was held between the thumb and the index finger during the experiment. The hand gripper had a U-shape and could be squeezed that, with increasing force, the two handles converged to each other (Figure 1). The hand gripper had a spring like behaviour, i.e. the closer the two handles of the gripper were brought the higher were the produced forces. The task consisted of pressing a hand gripper so that changes in the position of the hand gripper where proportional to changes of the force level. With a pressure of 10 N, the handles converged 8 mm to each other. A force transducer (Tekscan, Inc. South Boston, MA) was rigidly taped to the inside of the thumb during the whole experiment. Additionally, an angle goniometer (custom-built) was taped to the thumb and index finger to measure angle movements. In the force-controlled contractions the force was displayed on the screen and in the position-controlled trials the signal of the goniometer (position signal) was provided. Both signals were displayed in the same way as a running line on the monitor and were stored on a PC. The scale of the two types of feedback was adjusted so that movements between thumb and index finger resulted in the same deviation of the force and the position signal from the baseline. The gains of the two feedback signals were identical.

### EMG Recording

After preparation of the skin, bipolar surface electrodes (Blue sensor P, Ambu, Bad Nauheim, Germany) were attached to the skin with 2 cm interelectrode distance. The reference electrode was placed on the olecranon of the same arm. The EMG recordings were amplified (×1000), bandpass filtered (10–1000 Hz) and sampled at 4 kHz. All data was stored on computer using custom-built software (LabView based, National Instruments, Austin, TX) for off-line analysis. In the fatiguing task (Protocol 1), EMG recordings of the non-dominant hand were taken from the first dorsal interosseous (FDI) muscle, the abductor pollicis brevis (APB) and the flexor pollicis longus muscles (FPL). Coactivation of muscles, a strategy to assist joint stabilization [22], was calculated in the following way [EMG (%EMGmax) for FDI/APB]. For protocol 2, EMG recordings were obtained from the first dorsal interosseous muscle (FDI) of the non-dominant hand.

### Transcranial magnetic stimulation (TMS)

Magnetic stimuli were applied over the right motor cortex using a Magstim Rapid Rate Stimulator (Magstim Company Ltd., Whitland, UK) with a figure of eight coil (Magstim SP 16097). For each subject, the initial stimulation point was set approximately 0.5 cm anterior to the vertex and over the midline. The final position for the stimulation was determined by moving the coil anterior and right from the vertex, while the MEP size of the FDI was monitored (induced current was posterior-anterior). Resting motor threshold was determined at the lowest intensity to evoke an EMP in a least three out of five sweeps. The optimal position for eliciting MEPs in the FDI with minimal intensity was marked on a cloth bathing cap worn by the subjects with a felt pen. The handle of the coil was fixed to a stand (Manfrotto, Italy) directly behind the subjects' chair. Both, the coil and the head, were fixed with a velcro® strip on the subject's head and the chair respectively. The coil position relative to the skull was checked several times during the experiment. TMS was applied during the position-controlled and the force-controlled muscular contractions (see procedure).

### Experimental procedure of Protocol 1

After preparation, each participant was instructed to perform 3 maximal voluntary contractions (MVC). The MVC trials consisted of a gradual increase in force from zero to maximum over a 3 s time span. The maximal force was held for 2 to 3 s and subjects were verbally encouraged to achieve maximal force. After each trial there was a rest of 90 s. The peak force achieved in the 3 trials was considered as the MVC force value. In the subsequent trials, 30% of the peak force was taken as the force value during the position or the force-controlled contraction. The 30% MVC-value was represented by a red line on the computer screen and subjects were asked to meet this line with a black line corresponding to the actual exerted force produced by pressing the hand gripper. Alternatively, not the force but the position was displayed on the screen and subjects had to maintain the thumb-to-index finger angle, which corresponded to the individual thumb-to-index angle when subjects matched the force level of 30% MVC. The order of the sustained force or position-controlled contractions was counterbalanced and there was a break of at least 3 days between the measurements. Subjects were not informed about their time to task failure until completion of the two experimental sessions. The six subjects who participated in protocol 1 were instructed to sustain a position or force-controlled contraction at 30% of their MVC until task failure. Task failure was determined as when subjects were not able to hold the force within 5% of the target force for 5 s or when they were not able to keep the thumb-to-index finger angle within 5% of the target angle for 5 s. The required target force level and target finger angle were displayed on a computer screen placed 1 m in front of the subject.

### Experimental procedure of Protocol 2

The experimental setup was the same as in protocol 1. For protocol 2, subjects had to contract with 20% of their MVC. The lower contraction intensity compared to the fatiguing task was chosen to avoid any fatigue related bias. Furthermore, Seifert et al. [23] demonstrated that a contraction intensity of 40% MVC resulted in the same amount of EMG suppression caused by subTMS than lower contraction intensities. In the present study, subTMS was applied with a randomized interstimulus interval ranging from 0.8 to 1.1 seconds during the muscular contractions with position and force feedback. To analyze whether TMS caused a facilitation or suppression in the FDI EMG, the rectified and then averaged 40 sweeps without stimulation (control EMG) were subtracted from 40 sweeps with stimulation (see also Davey et al. [16], Petersen et al. [17] and Zuur et al. [18]). This means that each position and force-controlled contraction had to be maintained for approximately 80 seconds. The initial magnetic stimuli were always chosen to be high enough to evoke MEPs in the FDI. After one trial with position and one with force feedback executed at the same stimulus intensity, subjects were asked to relax and the stimulation intensity was then gradually decreased before the next trial started after a pause of 2 min. In this manner, the stimulus intensity was further decreased until a suppression of the EMG was visible without the presence of any facilitation. This adjustment served to reveal the maximal suppression by subTMS in both conditions. Finally, the stimulus intensity was further decreased until no difference between the averaged sweeps with and without stimulation could be observed. The trials were executed in blocks meaning that one trial with force feedback and one with position feedback were executed at the same stimulus intensity, but the order of the force and position-controlled contractions in each block was randomized to account for other variables (e.g. fatigue), which might otherwise have biased the results. Both trials were tested in one session and the subjects were aware whether they will have to perform a position or a force-controlled contraction.

### Data Analysis and Statistics

For protocol 1, maximal EMG activity (EMGmax) was calculated as the root mean square value taken over a 0.5 s interval around the rectified maximal EMG amplitude (EMGmax) obtained during the MVC. The EMGmax was assessed during the same experimental session with the identical setup in advance of the fatiguing contractions. During the sustained contraction, muscular activity was quantified by root mean square values of the rectified EMG over 8 s measured every 30 s during the course of the sustained contractions and normalized to EMGmax. To compare changes in EMG activity, the first 8 seconds of the sustained contraction were compared with the last 8 seconds. For protocol 2, the onset of the EMG suppression caused by subTMS was defined as the instant where the averaged EMG for the stimulated condition was less than the control EMG for at least 4 ms in a time window from 20 to 50 ms after the TMS. The end of the suppression was defined as the instant when the stimulated EMG was above the control EMG for more than 1 ms. The mean suppression was expressed as percentage change (control-stimulated)/mean_control_*100). The maximal suppression was defined as the instant with the greatest difference between the ensembled averages of the stimulated and control trial. Accordingly, the maximal suppression for the force-controlled and for the position-controlled contractions were individually calculated. The control EMG was averaged in the time window of analyzes. Peak to peak amplitude of the MEP was measured in a window of 50 ms for each stimulus.

Before comparing the variables, normal distribution of the data was tested using the Kolmogorov-Smirnov test. All data are reported as means ± standard deviation. For protocol 1 and 2, all statistical comparisons were made using Bonferroni-corrected two-sided paired *t*-tests. For protocol 1, a three-factor ANOVA (task×muscle×time) with repeated measures on all factors was calculated to compare EMG values of the FDI, APB and FPL for the two tasks [24]. SPSS 17.0 software was used for statistical analyses (SPSS®, Chicago, IL.). The level of significance was set at P≤0.05.

## Results

### Protocol 1: Time to task failure

**Figure 2:**
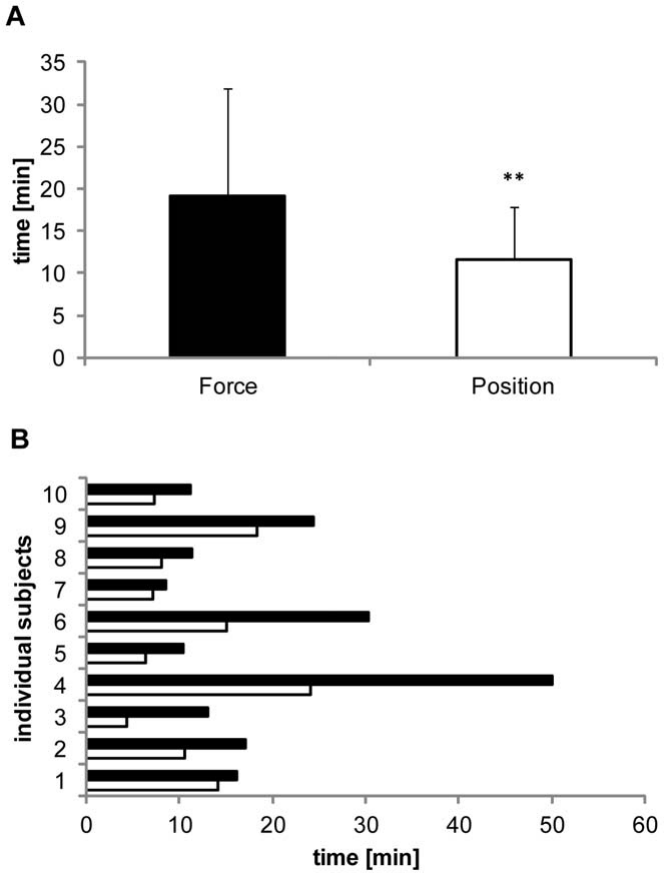
Times to task failure of sustained contractions. *A:* Mean endurance times for the force and the position-controlled contractions. The endurance time was significantly longer in the force-controlled contraction compared to the position-controlled contraction (**P = 0.01). *B:* Endurance times of the individual subjects. All subjects show longer endurance times during force control (black bar) than during position control (white bar).

All subject participating in protocol 1 displayed a significantly longer time to task failure in the force-controlled task (19.3±12.8 min) compared to the position-controlled task (11.5±6.3 min; P = 0.01; Figure 2). There was no difference in MVC values between the two tasks (force: 41.1±10.7N, position: 42.0±10.9N, P = 0.68). Analyses also revealed a significant time×muscle effect; F_2,18_ = 4.7; η^2^ = 0.2, P = 0.015).

### EMG activity

There was a strong trend towards an increased EMG activity during the fatiguing contractions (time main effect; F_1,9_ = 9.07; η^2^ = 0.4, P = 0.07) but there was no difference between the tasks (task×muscle; F_2,18_ = 2.5; η^2^ = 0.4, P = 0.87).

**Figure 3:**
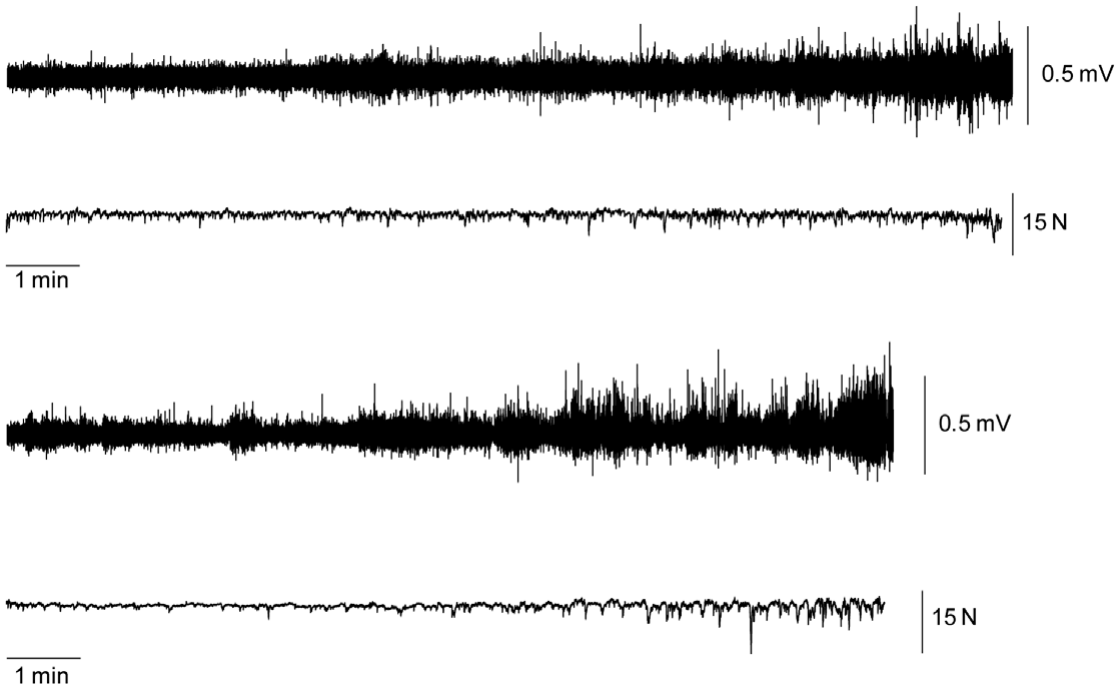
Increase in EMG activity in the course of the sustained contractions. Raw FDI EMG and force signals taken from a single subject for the force task (top panel) and the position task (bottom panel). There was a significant increase in EMG amplitude in the force and the position-controlled task.

The FDI EMG activity increased with time (time main effect; F_1,9_ = 16.0; η^2^ = 0.4, P = 0.01) in both the force (15.1±9.9% to 30.9±20.3% EMGmax) and the position-controlled task (16.3±12.01% to 29.3±20.2% EMGmax). The amount of increase in FDI EMG did not differ between the tasks (task×time; F_2,18_ = 1.0; η^2^ = 0.05, P = 0.34; Figure 3).

The APB EMG did not change with time (time main effect; F_1,9_ = 2.1; η^2^ = 0.1, P = 0.16) in both the force (22.9±21.9% vs. 24.7±23.0% EMGmax) and the position-controlled task (29.5±19.2% vs. 35.1±27.7% EMGmax). The APB EMG did not differ between the tasks (task×time; F_2,18_ = 1.1; η^2^ = 0.05, P = 0.31).

The FPL EMG did not change with time (time main effect; F_1,9_ = 4.7; η^2^ = 0.2, P = 0.33) in both the force (16.5±11.5% vs. 19.8±8.7% EMGmax) and the position-controlled task (14.1±6.9% vs. 18.8±10.7% EMGmax) and did not differ between the tasks (task×time; F_2,18_ = 0.1; η^2^ = 0.01, P = 0.79).

The coactivation ratio [EMG (%EMGmax) for FDI/APB] did also not change during the fatiguing tasks (force task, start: 1.59±1.69, end: 2.22±2.04; position task, start: 0.77±0.68; end: 3.68±8.0; (task×time; F_2,18_ = 2.1; η^2^ = 0.05, P = 0.34).

### Protocol 2

**Figure 4:**
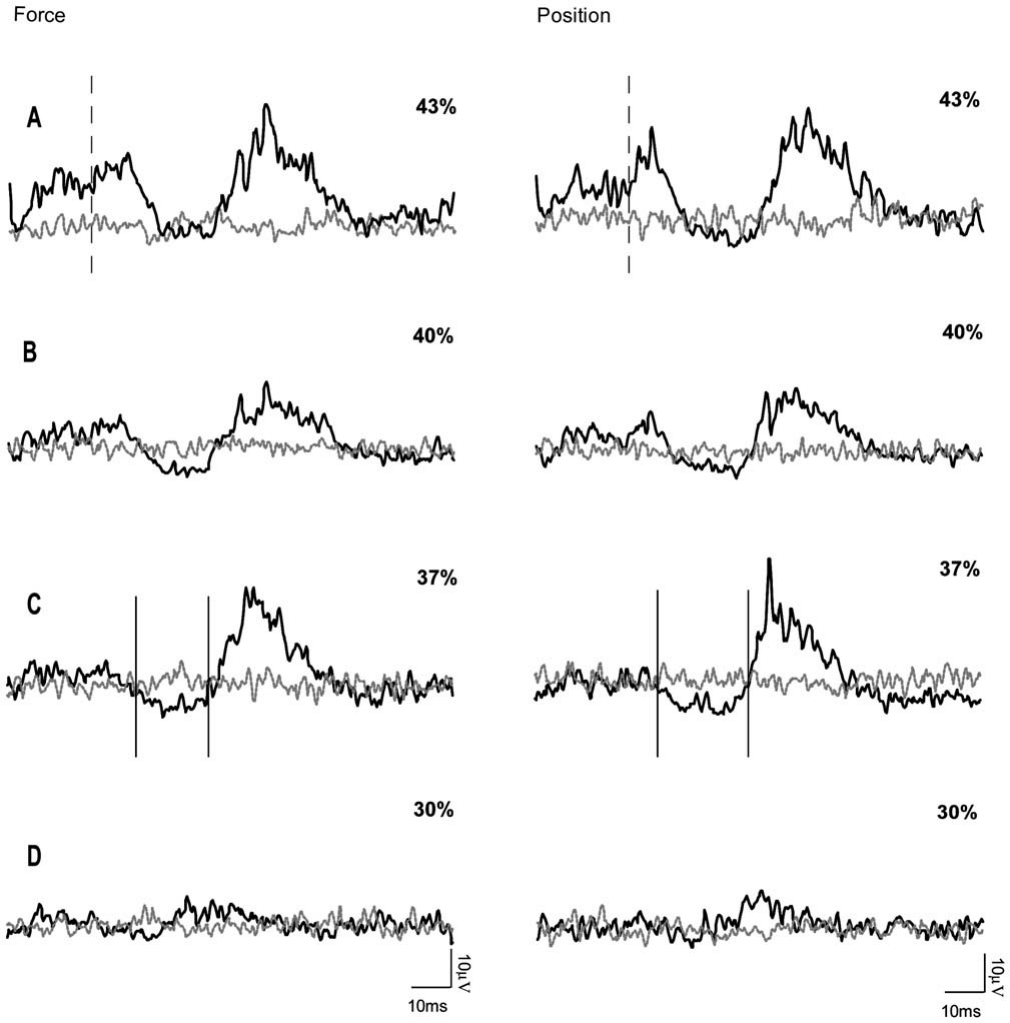
Development of EMG supression. Every trace is the average of 80 sweeps of the rectified FDI EMG. Traces are superimposed, the black line represents the average of sweeps with stimulation whereas the grey line represents the average EMG activity without stimulation. The sweeps shown with and without transcranial magnetic stimulation were randomly assessed. In this example of a single subject, four different stimulus intensities were used and are expressed as percentage of maximum stimulator output: *A* 43%; *B* 40%; *C* 37% and *D* 30%. The vertical dashed lines in *A* represent the onset of the facilitation and the vertical lines in *C* show the onset and end of the suppression. This time window was used in order to quantify the suppression. The left EMG traces represent the force-controlled contraction and the right panel the position-controlled contraction.

**Figure 5:**
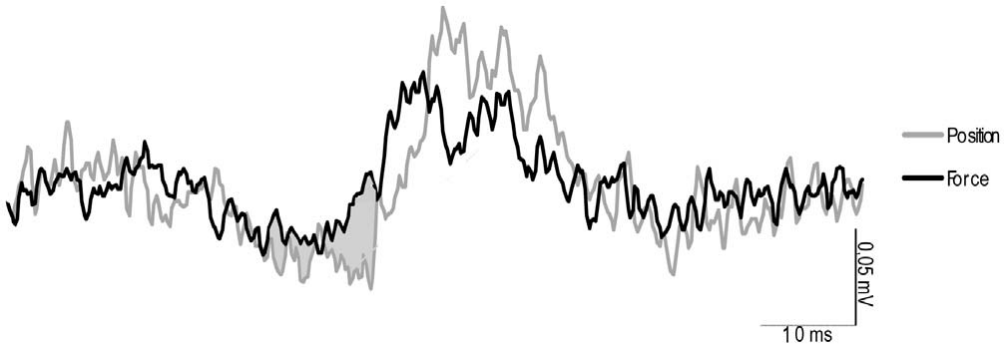
EMG suppression in one representative subject. Ensemble averaged FDI EMG taken from a single subject during the stimulated condition. The grey shaded area between the two traces indicates the differences in TMS-evoked suppression between the two tasks. The onset of the EMG suppression was similar but the extend was significantly greater in the position-controlled task.

**Figure 6:**
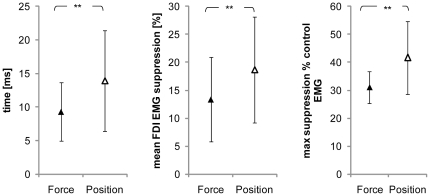
Group mean data of EMG suppression. Grand mean values (+SD) of the maximal EMG suppression in the force (▴) and the position-controlled task (▵). The maximum was greater in the position-controlled task compared to the force-controlled task (**P = 0.028) (A). Additionally, the duration of FDI EMG suppression caused by subTMS lasted significantly longer than in the force-controlled task (**P = 0.024) (B). Finally, the mean suppression of FDI EMG as percent of the control EMG. The FDI EMG suppression was significantly greater for the position-controlled task than for the force-controlled task (**P = 0.025) (C).

In all ten subjects who participated in this experiment, the use of subTMS to the motor cortex resulted in a significant suppression of the FDI EMG during the position-controlled and the force-controlled tasks. Figure 4 shows data from a single subject, demonstrating that at stimulation intensities above the motor threshold (43% Maximum Stimulator Output (MSO)) a clear MEP was observed (Figure 4 *A*). By constantly decreasing the stimulus intensity, the MEP (40% MSO) became first smaller (Figure 4 *B*), then resulted in a clearly visible suppression (Figure 4 *C*, 37% MSO) until finally no suppression of the FDI EMG was present any longer (Figure 4 *D*, 30% MSO). In this exemplary subject as well as in the data of all other subjects, there were no differences in the MEPs measured during the position-controlled and force-controlled contraction with suprathreshold stimulation (group mean: force 40±0.36 mA vs. position 42±0.31 mA; P = 0.71). The latency of the onset for the facilitation (MEP) visible at suprathreshold stimulation was 18.8±2 ms in the force-controlled task and 20.1±1.6 ms in the position-controlled task. The suppression of the FDI EMG caused by subTMS during the muscular contraction obtained at the same stimulus intensity was greater with position feedback than with force feedback. Figure 5 gives an example of a TMS evoked suppression of the FDI EMG from a single subject. When looking at the grand mean values of the FDI EMG suppression, a clear difference in the duration, mean and maximal suppression between the two tasks was observed. In detail, the duration in the position-controlled task lasted 13.9±7.5 ms compared to only 9.3±4.3 ms in the force-controlled task (F = 7.3; P = 0.024; Figure 6 *A*). There was no significant delay between the onset of the suppression in the position-controlled task and the onset of the suppression in the force-controlled task (force 29±3 ms; position 28±3 ms). For the contraction with force feedback, the mean suppression was on average only 13.3±7.5% and for the contraction with position feedback 18.6±9.4% of the control EMG (F = 7.2; P = 0.025) (Figure 6 *B*). Again, the maximal suppression was only 31.1±5.8% with the force feedback compared to 41.6±13.1% with the position feedback (F = 6.8; P = 0.028) (Figure 6 *C*). Furthermore, to minimize a potential bias induced by fatigue, EMG activity as well as force fluctuations of the first 40 sweeps and the second 40 sweeps within one trial were compared. No significant differences could be observed (force: p = 0.29, EMG: p = 0.13; position: p = 0.86, EMG: p = 0.23). To test whether the short interstimulus intervals of the subthreshold TMS had an effect on motor cortical output (i.e., to ensure that the short interstimulus intervals did not act like a train of repetitive TMS), the first 10 stimuli were compared with the last ten stimuli. Results revealed no significant effects (force: p = 0.64; position p = 0.64). Furthermore, EMG of the initial trial of one task (force or position) were compared with the values obtained in the last trial when providing the same kind of feedback. This comparison also did not reveal any differences (force: p = 0.11; position: p = 0.36).

The RMS EMG (sweeps without TMS) was analyzed over the same time span as the trial with stimulation and showed no differences between the two tasks (force = 0.06±0.04 mV; position 0.06±0.03 mV; F = 9.215; P = 0.591).

## Discussion

The purpose of the present study was to evaluate whether the presentation of different feedback modalities influences the time to task failure of a sustained fatiguing contraction. The results show a significantly reduced time to exhaustion when the subjects were provided with feedback about their joint position instead of the applied force. Because the mechanics of the two tasks were identical resulting in a comparable EMG increases, we speculated that there might exist profound feedback depended differences at the cortical level, too. This assumption could be supported due to the presence of a greater mean and maximal EMG suppression after subTMS in position controlled contractions.

### Functional implications

In a number of studies, Enoka and coworkers compared two different contraction types controlled via external feedback. The first one involved contractions in a compliant (non-rigid) system and the subjects received visual feedback about their position (position task). The second contraction was executed in a non-compliant, rigid system and subjects received feedback about their force (force task; see [25]). The subjects displayed shorter time to task failure when they received feedback about their joint position than when force feedback was given [25]. However, the present results can hardly be compared with data of these previous studies, as those studies did not only alter the type of feedback but also the stiffness of the experimental device [26]–[31]. Thus, during position-controlled contractions subjects worked against a compliant system whereas in force control the system was rigid (non-compliant) [26]–[31].

The results of the current study show that the time to task failure of the fatiguing contractions was also significantly prolonged in the force compared to the position-controlled contraction. Thus, the data has some similarity with the results obtained by the group around Enoka. However, the difference in time to task failure in our tasks can clearly be attributed to the different kind of feedback as the mechanics were identical. Therefore, the shorter time to task failure associated with position control despite identical mechanics of the tasks may indicate that position-controlled contractions are differently controlled compared to force-controlled contractions. This might be due to differences in the motor control of this movement and/or a differential neural processing of the afferent feedback associated with force and position controlled contractions.

### Changes in motor cortical activity

The purpose of the second part of our study was to evaluate whether motor cortical activity differs in position-controlled contractions compared to force-controlled contractions. To highlight differential activity of the motor cortex during the two tasks, subTMS was applied in order to inhibit voluntary EMG activity by suppressing the cortical output [16], [17]. M1 was chosen because it is part of the transcortical (reflex) loop [12], [13] and being a key junction for voluntary control [14], [15]. The results demonstrate that despite comparable mechanical properties of the position and the force-controlled contractions, subTMS suppressed the EMG to a greater extent in the position-controlled task. This indicates differences at the motor cortical level with respect to the type of feedback in the non-fatigued muscle.

Milner and Hinder [11] suggested that position rather than force information is used to adapt to changes in the environmental dynamics even though this might not always be the optimal strategy. The authors speculated that peripheral load sensors like the Golgi tendon organs might not be able to unambiguously signal the direction of the force field as a change in the force-field direction or in the force-field strength would unload the tendon organs in the same way. The force field described in the study by Millner and Hinder can be understood as an area where several force points with varying strength produce a force lateral to the hand movement. In contrast to their study, the mechanical environment in our study remained unchanged. Thus, task-related differences in cutaneous or proprioceptive feedback are unlikely and can therefore not explain the observed differences between force and position-controlled contractions. The disparity in EMG suppression after subTMS might therefore more likely be explained by differential central processing of force and position feedback. The feedback-specific processing in M1 might also explain – at least partly – the behavioral differences between these two tasks, which were highlighted by the observation of differences in the time to task failure.

Previous fMRI experiments reported that increasing the movement precision in motor tasks caused an enhanced regional cerebral blood flow in the primary and non-primary motor cortices [32]. In line with this, Pearce and Kidgell [33] reported greater MEPs in the task requiring enhanced movement precision indicating an enhanced corticospinal excitability. According to these two studies it seems that the biomechanics of the task determine the involvement of the motor cortex. Complementary to this assumption, Seifert & Petersen [23] demonstrated that the amount of EMG suppression did not depend on the force level when subjects performed sustained contractions of the elbow flexors at various contraction intensities raging from 10% MVC up to 40% MVC. Like in the current study, the authors used subTMS to reveal the motor cortical activity. SubTMS produced no greater EMG suppression when the contraction intensity was increased from 10 to 20, 30, and finally 40% MVC in the non-fatigued muscle. Thus, it seems that not the intensity but rather the cognitive demands of the task determine the amount of EMG suppression. However, another finding of this study was that the amount of subTMS evoked EMG suppression increased during the development of fatigue [23]. Therefore, it could be argued that fatigue might have been responsible for the differences in EMG suppression observed in the current study. However, the comparison of the first and second half of the 80 sweeps and the comparison of the initial with the last trials did not reveal any differences. Additionally, blocks of 80 stimuli were recorded for each task (force and position) in a randomized order. Thus, fatigue would have affected both tasks in a very similar way. Furthermore, the order of force and position-controlled tasks was individually randomized.

Besides the finding that the motor cortical activity was different between position- and force-controlled contractions, the exact mechanisms within the cortex remain unknown: One likely explanation for our results is that subTMS had a greater effect in position-controlled contractions because the motor cortical output might be greater in this task compared to the force task. Another explanation for the differences in the EMG suppression in force and position-controlled tasks is the activation of different intracortical interneurons with different locations. Although inhibitory GABAergic neurons are present throughout the cortex [34], it might at least theoretically be the case that the inhibitory intracortical neurons, which have been activated by subTMS during the force-controlled task, lie deeper within the cortex.

The results of the present study show a reduced time to task failure and an increased TMS evoked EMG suppression during a position-controlled contraction compared to a force-controlled contraction. Together with the results obtained in earlier studies, the present results further support the notion that position and force-information is differently organized and/or integrated in the CNS and that this has also functional consequences (shown by a shorter time to task failure in the position-controlled task). We cannot directly link the electrophysiological data and the data obtained during the fatiguing task, but it might be that the differences in cortical activity contribute to the differences in time to task failure. Based on our results it might be worthwhile to investigate the influence of force and position-controlled exercises in rehabilitation as different neural circuits are probably activated. Furthermore, sport disciplines primarily relying on position control, like archery for instance, may benefit from the application of position-controlled strength exercises instead of the force-controlled tasks that are used nowadays.
